# Impact of lignin polymer backbone esters on ionic liquid pretreatment of poplar

**DOI:** 10.1186/s13068-017-0784-2

**Published:** 2017-04-20

**Authors:** Kwang Ho Kim, Tanmoy Dutta, John Ralph, Shawn D. Mansfield, Blake A. Simmons, Seema Singh

**Affiliations:** 1 Deconstruction Division, Joint BioEnergy Institute, Emeryville, CA USA; 2 Biological and Engineering Sciences Center, Sandia National Laboratories, 7011 East Avenue, Livermore, CA 94551 USA; 3 Department of Biochemistry, University of Wisconsin, Madison, WI USA; 4 Department of Energy Great Lakes Bioenergy Research Center, Wisconsin Energy Institute, Madison, WI USA; 5 Department of Wood Science, University of British Columbia, Vancouver, Canada; 6 Biological and Engineering Sciences Division, Lawrence Berkeley National Laboratory, Berkeley, CA USA

**Keywords:** Biofuels, Biomass, Lignocellulose

## Abstract

**Background:**

Biomass pretreatment remains an essential step in lignocellulosic biofuel production, largely to facilitate the efficient removal of lignin and increase enzyme accessibility to the polysaccharides. In recent years, there have been significant efforts *in planta* to reduce lignin content or modify its composition to overcome the inherent recalcitrance that it imposes on lignocellulosic biomass during processing. Here, transgenic poplar lines in which monolignol ferulate conjugates were synthesized during cell wall development to introduce, during lignification, readily cleavable ester linkages into the lignin polymer backbone (i.e., “zip lignin”), along with wild-type (WT) controls, were pretreated with different ionic liquids (ILs).

**Results:**

The strategic introduction of ester bonds into the lignin backbone resulted in increased pretreatment efficiency and released more carbohydrates with lower energy input. After pretreatment with any of three different ILs, and after limited saccharification, the transgenic poplars, especially those with relatively higher amounts of incorporated monolignol ferulate conjugates, yielded up to 23% higher sugar levels compared to WT plants.

**Conclusion:**

Our findings clearly demonstrate that the introduction of ester linkages into the lignin polymer backbone decreases biomass recalcitrance in poplar has the potential to reduce the energy and/or amount of IL required for effective pretreatment, and could enable the development of an economically viable and sustainable biorefinery process.

**Electronic supplementary material:**

The online version of this article (doi:10.1186/s13068-017-0784-2) contains supplementary material, which is available to authorized users.

## Background

In recent years, lignocellulosic biomass has received a significant attention as a renewable resource to reduce societal dependence on fossil fuels. It is estimated that there are over one billion tons of biomass available annually in the United States alone for conversion into biofuels and renewable chemicals [1, 2]. Moreover, the adoption of near-term technologies, such as cellulosic ethanol, could effectively reduce net automotive greenhouse gas emissions [3]. From a techno-economical standpoint, however, the relatively high production cost of cellulosic ethanol has thus far limited its commercialization, compared to the less favorable but technically uncomplicated first-generation ethanol from cane sugar or corn grain starch. In the biomass-to-ethanol process, pretreatment has consistently been highlighted as one of the most expensive unit operations, which has largely been attributed to the recalcitrance of the lignocellulosic cell wall structure. Among the three biomass components, the lignin polymer acts as the major impediment to bioethanol processing [4].

There have been many efforts to redesign lignin to render it more amenable to chemical depolymerization, which can lower the energy required for industrial processing [4–7]. For example, switchgrass recalcitrance has been reduced by downregulating the *caffeic acid 3*-*O*-*methyltransferase* (*COMT*) gene in the lignin pathway; a reduced lignin content and altered lignin composition led to increases in saccharification efficiency, requiring less severe pretreatment and lower enzyme loading to achieve the same ethanol yield [7]. Similarly, poplars downregulated in *cinnamoyl*-*CoA reductase* (*CCR*) had more facile saccharification but at a biomass yield penalty [8]. A selective reduction in lignin and increase in cellulose accumulation was shown to impact the secondary cell wall structure in *Arabidopsis* [9]. Recently, with the basic idea of introducing readily cleavable bonds into the lignin backbone to lower the energy requirement for biomass processing, poplar was engineered to produce monolignol ferulate conjugates to augment the monomer pool by introducing an exotic *feruloyl*-*CoA monolignol transferase* (*FMT*) genes [4]. The resultant generation of lignin polymer backbone chains containing ester linkages improved cell wall digestibility, liberated more sugars after pretreatment, and improved saccharification compared to the WT [4].

Among the different pretreatment approaches, certain ILs have shown considerable promise as efficient and effective solvents for biomass pretreatment. ILs are molten organic salts composed of ions, and most of them exist in the liquid state below 100 °C [10]. Moreover, pretreatments employing ILs can occur under milder conditions, and ILs can be recycled and reused after biomass processing [11]. IL pretreatment can preferentially remove lignin and convert microcrystalline cellulose to its non-crystalline allomorph [12–14]. For example, 1-ethyl-3-methylimidazolium acetate ([C_2_C_1_Im][OAc]) has been found to completely dissolve biomass under specific pretreatment conditions, providing a promising platform to separate the biomass components [2]. Recently, ILs containing choline cations and amino acid anions [Ch][AA], coined “bionic liquids”, have been shown to selectively remove lignins, resulting in high sugar yields [15–17]. Along with their lignin dissolving efficiencies, bionic liquids have gained significant favor because of their biocompatibility with enzymes and even with microorganisms, especially when compared to imidazolium-based ILs [15], opening up the possibility of a one-pot process for ethanol production [18]. Pretreatment efficiency studies employing imidazolium- and choline-based ILs found that [Lys]^−^ anions could provide greater delignification and higher glucose yields than ILs containing [OAc]^−^ anions [15]. In addition to the bionic liquids, recent development of a highly effective pretreatment process utilizing the aqueous IL composed of tetrabutylammonium and hydroxide ions ([TBA][OH]) generated high glucose yields after pretreatment under mild conditions (50 °C) [19]. The [TBA][OH] IL was found to have higher ionic mobility, enhancing the removal of lignin and noncellulosic components of biomass under mild pretreatment conditions [19].

We have previously reported on the development of IL pretreatment processes using various ILs [2, 15, 19–21]. Herein, we demonstrate that utility of ILs for pretreating transgenic poplar whose lignin was designed for improved deconstructability by introducing chemically labile esters into the lignin backbone. We show that the transgenic poplar required less severe pretreatment and can be processed more readily to liberate significantly more sugar monomers under milder pretreatment conditions.

## Methods

### Raw materials

Transgenic poplar (*Populus alba* × *grandidentata*) that was originally developed and grown at the University of British Columbia, Vancouver, BC, Canada, was provided by the Great Lakes Bioenergy Research Center (GLBRC) [4]. Briefly, hybrid poplar transformed with an exogenous *feruloyl*-*CoA monolignol transferase* (*FMT*) that catalyzes the formation of monolignol ferulates that ultimately get incorporated into the lignin polymer during lignification were used for all experiments. The details, including gene identification and expression analysis (*FMT*), plant growth, and compositional analysis, have been previously described [4]. Both transgenic and WT poplars were ground and sieved to a constant size of 1–3 mm prior to pretreatment. 1-Ethyl-3-methylimidazolium acetate [C_2_C_1_Im][OAc] was purchased from BASF (Florham Park, NJ) and used as received. Cholinium lysinate [Ch][Lys] and tetrabutylammonium hydroxide [TBA][OH] were purchased from Iolitec (Tuscaloosa, AL) and used as received. The commercial cellulase mixture (Cellic^**®**^ CTec2) and hemicellulase mixture (Cellic^**®**^ HTec2) were generously provided by Novozymes (Davis, CA) and used as received. Compositional analysis of raw materials and pretreated poplar samples was conducted using a National Renewable Energy Laboratory (NREL) protocol [22].

### Biomass pretreatment and saccharification

A 10 wt% biomass solution was carefully prepared by combining 0.2 g (dry weight) of biomass with 1.8 g of one of three ILs in a 10 mL pressure tube (Ace Glass, Vineland, NJ). The tubes, in triplicate, were heated in an oil bath to a predetermined temperature with continuous stirring (Table 1). After pretreatment, 6 mL of deionized (DI) water was added to the reaction slurry, and the mixture transferred to 50 mL Falcon tube that was then centrifuged at 1000 rpm to separate the lignin from the supernatant containing the ILs. The pelleted precipitates, mainly polysaccharides, were then washed four times with 6 mL of DI water (4 × 6 mL) to remove any residual IL.

**Table 1:** Ionic liquids used in this study, and pretreatment conditions studied

ILs	Structure	Pretreatment conditions
[C_2_C_1_Im][OAc]		Temperature: 160 °C Time: 3 h Biomass loading: 10 wt%
[Ch][Lys]		Temperature: 140 °C Time: 1 h Biomass loading: 10 wt%
[TBA][OH]		Temperature: 70 °C Time: 3 h Biomass loading: 10 wt%

Enzymatic saccharification of the pretreated biomass was conducted for 72 h in 5 mL of 50 mM sodium citrate buffer (pH 4.8) supplemented with Cellic^**®**^ CTec2 and HTec2 (188 mg/mL) from Novozymes (9:1 ratio) at 10 mg protein/gram solid biomass of, at 50 °C and 150 rpm in a rotary incubator.

### Analytical methods

After pretreatment and saccharification, the hydrolysate was separated from the residual substrate by centrifugation, and then filtered through a 0.45 μm syringe filter. Glucose and xylose release was quantified using an Agilent 1100 series high-performance liquid chromatograph (HPLC) equipped with a Bio-Rad Aminex HPX-87H ion-exchange column and a refractive index (RI) detector. The mobile phase employed to achieve separation was 4 mM H_2_SO_4_ at a constant flow rate of 0.6 mL/min and the column temperature was maintained at 60 °C. The yield of glucose and xylose was determined after subtracting their levels in an enzyme blank.

Gel-permeation chromatography (GPC) was used to determine the relative molecular weight distribution of the lignin-rich residue after enzymatic saccharification. GPC measurements were carried out using a Tosoh Ecosec HLC-8320 GPC equipped with a UV detector, whose wavelength was set at 280 nm. The eluent for the analysis was tetrahydrofuran and the column used was an Agilent PLgel 3 μm 100 Å (300 × 7.5 mm). The column flow rate was 1.0 mL min^−1^ at 35 °C. Polystyrene standards were purchased from Agilent (Agilent Technologies, Inc., Santa Clara, CA) and used to establish a calibration curve that ranged from 162 to 29,150 g mol^−1^. All samples were derivatized using acetic acid and acetyl bromide (92:8, v/v) for 2 h at 60 °C prior to GPC analysis [23].

Raw biomass and residual solids from WT and transgenic Line 5 after IL pretreatment were ball-milled, solubilized in 4:1 DMSO-*d*_*6*_/pyridine-*d*_*5*_, and then analyzed by two-dimensional (2D) ^1^H–^13^C heteronuclear single-quantum coherence (HSQC) nuclear magnetic resonance (NMR) spectroscopy as previously described [24, 25]. In short, samples were ball-milled using a Fritsch Pulverisette 7 and approximately 50 mg were placed in NMR tubes with 600 μL DMSO-*d*_*6*_/pyridine-*d*_*5*_. The samples were sealed and sonicated until homogeneity in a Branson 2510 table-top cleaner (Branson Ultrasonic Corporation, Danbury, CT). The temperature of the bath was closely monitored and maintained below 55 °C. HSQC spectra were acquired at 25 °C using a Bruker Avance-600 MHz instrument equipped with a 5 mm inverse gradient ^1^H/^13^C cryoprobe using the “hsqcetgpsisp2.2” pulse program (ns = 200, ds = 16, number of increments = 256, d1 = 1.0 s). Chemical shifts were referenced to the central DMSO peak (*δ*_C_/*δ*_H_ 39.5/2.5 ppm). Assignment of the HSQC spectra is described elsewhere [24, 26]. Changes in lignin structural characteristics were determined based on volume integration of HSQC spectral contour correlations using the Bruker’s Topspin 3.1 processing software.

### Statistical analysis

Analysis of variance (ANOVA) was conducted using R (R Foundation for Statistical Computing, Vienna, Austria) to test the null hypothesis of no statistical differences in saccharification yield between the WT and transgenic FMT poplar. Triplicate samples were collected for each transgenic line and the corresponding control trees. The null hypothesis was rejected at the 0.05 level.

## Results

### Pretreatment of poplar with cholinium lysinate ([Ch][Lys])

**Fig. 1:**
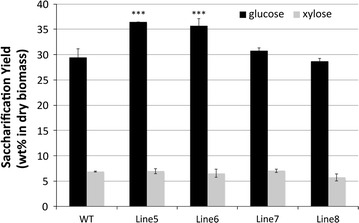
Saccharification yield (wt% based on initial dry biomass) from the [Ch][Lys] pretreated WT and transgenic poplars (statistical significance from WT ***P < 0.001; **P < 0.01; *P < 0.05)

Pretreatment of transgenic poplar with [Ch][Lys] was carried out at 140 °C for 1 h, a pretreatment condition previously determined to be optimal for switchgrass [15]. The pretreated substrates were then hydrolyzed using commercial enzymes, at 50 °C and a pH of 4.8. As shown in Fig. 1, significant increases in saccharification yield after [Ch][Lys] pretreatment were apparent in some transgenic poplar lines, especially Lines 5 and 6. Under these conditions, the yield of glucose (on a dry initial biomass basis) released from WT was 29.5%, whereas 36.4% (23.4% higher) glucose was liberated from Line 5. The amount of ML-FA conjugate incorporated during poplar lignification can only be crudely estimated from the low release of diagnostic conjugates [4, 27], but digestibility is hypothesized to be associated with the level of ML-FA conjugate in the lignin. The trend observed in the digestibility efficiency in this study is consistent with previous saccharification results that used mild alkaline pretreatments [4]. Compared to mild alkaline pretreatment, however, the overall glucose yield was increased approximately 40% when pretreated with [Ch][Lys]. The higher sugar yield is likely associated with the performance of [Ch][Lys] as a pretreatment. The strategic introduction of ester bonds into the lignin backbone did not affect xylose yield, likely due to the poor xylan recovery after pretreatment [15]. As shown in the mass balance analysis (see Additional file 1), only 52–61% of xylan was actually recovered.

### Pretreatment of poplar with 1-ethyl-3-methylimidazolium acetate ([C_2_C_1_Im][OAc])

**Fig. 2:**
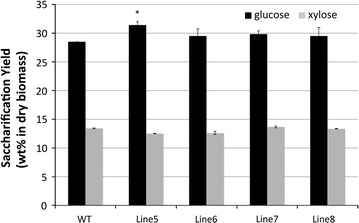
Saccharification yield (wt% based on initial dry biomass) from the [C_2_C_1_Im][OAc] pretreated WT and transgenic poplars (statistical significance from WT ***P < 0.001; **P < 0.01; *P < 0.05)

Imidazolium-based ILs, such as [C_2_C_1_Im][OAc], have been recently evaluated for their use in pretreatment processes. It was clearly shown that [C_2_C_1_Im][OAc] can effectively solubilize plant cell wall components, which then allows selective precipitation of cellulose by adding an anti-solvent, leaving a large fraction of the lignin in solution [28], and results in a readily hydrolysable polysaccharide fraction. Figure 2 shows the sugar yields after [C_2_C_1_Im][OAc] pretreatment followed by enzymatic hydrolysis. Again, the transgenic poplar lines yielded higher glucose. Among the transgenic lines, Line 5 showed the highest glucose yield, 10% higher than that of the WT poplar. Although the overall glucose yields are lower than those derived from the [Ch][Lys] pretreatment process, they were again higher for the transgenic poplar, which had a lower lignin content after pretreatment (see Additional file 1 for mass balance analysis). However, when the samples were pretreated with [C_2_C_1_Im][OAc], most of the xylan (>90%) was recovered, resulting in high xylose yields (which were not significantly different) after subsequent saccharification.

### Pretreatment of poplar with tetrabutylammonium hydroxide ([TBA][OH])

**Fig. 3:**
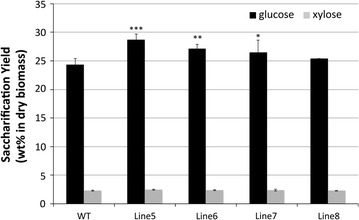
Saccharification yield (wt% based on initial dry biomass) from the [TBA][OH]-pretreated WT and transgenic poplars (statistical significance from WT ***P < 0.001; **P < 0.01; *P < 0.05)

Although [C_2_C_1_Im][OAc] shows significant cellulose dissolution, the IL and its solutions are relatively viscous and, therefore, require longer reaction times to dissolve the cellulosic fraction. Moreover, it is hard to handle, and even a small amount of water can cause a significant decrease in the cellulose solubility [29]. Recently, onium hydroxide solutions with water have been confirmed to dissolve cellulose at room temperature [30]. A hydrated IL [TBA][OH], with properties suggesting that it could be useful as a next-generation solvent for biomass pretreatment, was, therefore, also used to determine how well the cell walls containing the conjugates could be processed under mild conditions. Figure 3 shows the sugar yields from the [TBA][OH]-pretreated poplars. The glucose yields were again statistically higher with wood derived from the transgenic poplars (Lines 5, 6, and 7) than those from the corresponding WT. Similar to other IL pretreatments, transgenic Line 5 produced the highest glucose yield, approximately 17.7% higher than WT. However, the yields of xylose were less than 3% for all samples. As the mass balance analysis shows, less than 20% of the xylan was recovered after pretreatment.

### Impact of plant cell wall modification on pretreatment severity

**Fig. 4:**
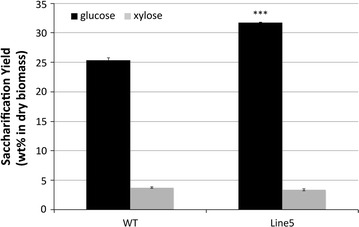
Saccharification yield (wt% based on initial dry biomass) from the [Ch][Lys] pretreated WT and transgenic Line 5 at 100 °C (statistical significance from WT ***P < 0.001; **P < 0.01; *P < 0.05)

It is clear that the transgenic poplar lines examined herein release more carbohydrates than the corresponding WT biomass when pretreated with any of the ILs tested. The use of [Ch][Lys] resulted in the best yields among the three ILs evaluated. It was logically hypothesized that the reduced recalcitrance of the transgenic poplar would facilitate the release of significantly more monosaccharides after pretreatments under lower severity conditions. To test this hypothesis, WT and the best overall performing transgenic line (Line 5) were pretreated with [Ch][Lys] at 100 °C. Under these conditions, Line 5 yielded 31.8% glucose versus only 25.4% from WT (Fig. 4). Although the yields were lower than those from pretreatments at 140 °C, the transgenic poplar again showed a significant increase in glucose release. This clearly demonstrates that employing trees with designer lignin can indeed decrease the inherent recalcitrance of biomass and has the potential to reduce the energy required for effective pretreatment.

### HSQC analysis of structural changes to lignin (and polysaccharides) during pretreatment

**Fig. 5:**
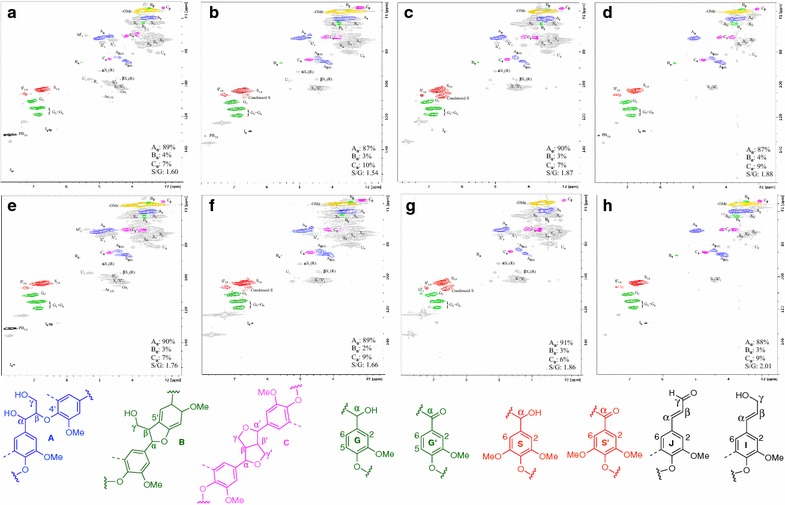
2D HSQC NMR spectra of WT and FMT-transgenic lignins. Wild-type (**a**) un-pretreated native material, and pretreated with (**b**) [Ch][Lys], (**c**) [C_2_C_1_Im][OAc], and (**d**) [TBA][OH]. Transgenic Line 5: (**e**) raw, and pretreated with (**f**) [Ch][Lys], (**g**) [C_2_C_1_Im][OAc], and (**h**) [TBA][OH]. **A**, β-ether (β-*O*-4′) unit; **B**, phenylcoumaran (β-5′) unit; **C**, resinol (β-β´) unit; **I**, hydroxycinnamyl alcohol endgroup; **J**, hydroxycinnamaldehyde endgroup; **PB**, *p*-hydroxybenzoate unit; G, guaiacyl unit; G′, benzyl-oxidized G unit; S, syringyl unit; S′, benzyl-oxidized S unit; X, xylan unit; X′, acetylated xylan unit; X(R), xylan including reducing end-unit; U, uronic acid unit; Ar, arabinan unit; Ga, galactan unit

The starting biomass and the corresponding IL-pretreated biomass were subjected to HSQC–NMR analyses to elucidate the effects of IL pretreatment on cell wall structure. The HSQC spectra, respective peak assignments, and substructures of WT and transgenic Line 5 are shown in Fig. 5. There were no distinct differences in the relative content of prominent lignin units, with their characteristic interunit linkages, or S/G ratios between the samples. The HSQC spectra can be divided broadly into well-resolved aromatic (*δ*_H_/*δ*_C_ 6.0–8.0/90–160) and aliphatic (*δ*_H_/*δ*_C_ 2.5–6.0/50–90) regions that contain the carbohydrate and lignin (oxygenated) aliphatic signals.

The aliphatic region provides important information about the various types of lignin units, characterized by their various interunit linkages. All HSQC spectra of raw and IL-pretreated biomass showed correlations corresponding to all of the sidechain C/H pairs for β-ether (β-*O*-4′, substructure **A**), phenylcoumaran (β-5′, substructure **B**), and resinol (β-β′, substructure **C**) units. The relative amounts of each are estimated from the volume integrals of the **A**_α_, **B**_α_, and **C**_α_ correlations, and expressed as a fraction of the total; the **C**-unit integrals are divided by 2, as a resinol unit contains two identical C/H pairs per unit. As is normal for hardwood lignins, both the WT and transgenic poplars were found to be rich in β-ether units **A** with marginal amounts of β-5′ and β-β′ units, and as is also normal from S-rich lignins, no signals were apparent from dibenzodioxocins that can result from the 5-5-coupling of G phenolic end-groups of oligomers [31].

The spectra of the pretreated biomass samples from the WT and transgenic poplars exhibit similar features for all three IL pretreatments. The β-*O*-4′ content of both WT and transgenic poplar was found to be marginally (but probably not statistically significantly) lower than the respective untreated biomass for [Ch][Lys] and [TBA][OH] pretreatments. The reduction in β-*O*-4′ content, which is measured by the volume integral of **A**_α_ correlation, may be a result of dehydration and the corresponding depolymerization that occurred during the pretreatment process.

The aromatic regions are dominated by the signals from the aromatic ring correlations from syringyl (S) units (derived from sinapyl alcohol) and guaiacyl (G) units (derived from coniferyl alcohol). S units show distinct signals from magnetically equivalent C_2,6_–H_2,6_ correlation, whereas G unit shows multiple signals corresponding to C_2_–H_2_, C_5_–H_5_, and C_6_–H_6_ correlations. Both the WT and transgenic poplar lignins are S-rich S–G lignins as in previously published results [4, 31, 32]. The S/G ratio for the transgenic may be slightly higher than for the WT. No distinct signals from *p*-hydroxyphenyl (H) units (derived from *p*-coumaryl alcohol) were observed at the shown contour level. Oxidized (α-ketone) structures from S and G units (i.e., S′ and G′) were identified in some of the samples. Apart from S and G units, signals from *p*-hydroxybenzoates (**PB**), unsaturated side-chains from cinnamaldehyde (**J**), and cinnamyl alcohol (**I**) end-groups (on S or G units) were also apparent. No signals corresponding to the monolignol ferulates that had been incorporated into the lignins could be observed in the transgenic samples, most probably because of the huge array of structures that can form, as noted in the supplementary information of the paper describing these transformed plants [4], and the consequent low abundance, below the NMR detection limit, of any such entities. Indeed, in currently unpublished work, it is strikingly difficult to discern discrete correlation signals even in cell wall model systems in which 20, 40, and 60% coniferyl ferulates have been introduced into coniferyl alcohol lignification, except at the highest levels.

A decrease in the **PB** content, which is logical from the ease of cleaving this terminal aromatic ester, was observed for all pretreated biomass, along with a slight decrease in the cinnamaldehyde **J** and cinnamyl alcohol **I** end groups. No distinct differences in the changes in the S/G ratio were observed between WT and transgenic poplar when subject to all three IL pretreatments, and both types of biomass exhibit similar trends. A slight decrease in S/G ratio was observed for [Ch][Lys] pretreated poplar, whereas a slight increase in the S/G ratio was observed for [C_2_C_1_Im][OAc] and [TBA][OH] pretreated poplar samples. The decrease in the S/G ratio for [Ch][Lys] pretreated poplar may be due to a loss of methoxyl units directly on S units, or to lignin depolymerization and subsequent dissolution into the IL. The [Ch][Lys] and [C_2_C_1_Im][OAc] pretreated poplars show signals corresponding to condensed S units, suggesting repolymerization reactions during the pretreatment.

The HSQC spectra also provided information on the polysaccharides present in the different samples. The anomeric region of the HSQC spectra contains the saccharide C_1_–H_1_ correlation signals. Different carbohydrate signals corresponding to xylans (e.g., X1–X′1, α-X1[R], and β-X1[R]), arabinans (Ar1[T]), galactans (Ga1), and glucuronic acid (U1) were also observed in this region of the HSQC spectra. In the aliphatic region, signals from *O*-acetylated xylans, namely, 3-*O*-acetyl-β-d-xylopyranoside (X′3) and 2-*O*-acetyl-β-d-xylopyranoside (X′2), can be clearly identified [24, 25].

The carbohydrate signals of the IL-pretreated biomass show some distinct differences. In the aliphatic region, the signals associated with two distinct peaks, X′2 and X′3, are significantly lower after [Ch][Lys] and [C_2_C_1_Im][OAc] pretreatment, and were absent after [TBA][OH] pretreatment. These findings suggest that hemicellulose deacetylation occurs during IL pretreatment. The deacetylation behavior in the various ILs is intriguing; in [Ch][Lys], the X3-OAc appears to have been somewhat selectively removed (Fig. 5b, f); in [C_2_C_1_Im][OAc], both are moderately well retained (Fig. 5c, g); and in [TBA][OH], complete xylan deacetylation has occurred (Fig. 5d, h). The anomeric regions of both biomasses also show noticeable decreases in the signals associated with of α-X1(R)/β-X1(R) that may be ascribed to glycosidic bond cleavage and reductions in the degree of polymerization (DP) of hemicellulose during IL pretreatment. Signals corresponding to xylans (β-d-xylopyranoside) were apparent for C_2_–H_2_ (X2), C_3_–H_3_ (X3), C_4_–H_4_ (X4), and C_5_–H_5_ (X5) correlations, which significantly overlaps with unassigned signals from pentose and hexose polysaccharide units, and were also less intense after IL pretreatment compare to those of the raw biomass in both WT and transgenic samples with maximum reduction observed for biomasses after [TBA][OH] pretreatment. Apart from these, signals from C_4_–H_4_ correlations of 4-*O*-methyl-α-d-glucuronic acid (U4) were also observed in some samples; these units are either well cleaved or ‘disappear’ with their attached xylan units under the various IL pretreatments.

In summary, none of the IL pretreatments caused obviously striking structural changes to the transgenics vs. their respective WT controls. However, the alterations to the residual cell wall materials following pretreatments were substantially different for the three ILs used. As compared to the raw material, the following are notable. (1) lignins are not significantly changed except that the apparent condensation is quite substantial, and PB esters are efficiently cleaved from the lignin with all ILs. (2) Deacetylation is intriguingly (and hugely) different with the 3 ILs. (3) There is significantly less hemicellulose retained in both WT and transgenic samples after [TBA][OH]-pretreatment.

### Molecular weight distribution of lignin residues obtained after enzymatic saccharification

**Fig. 6:**
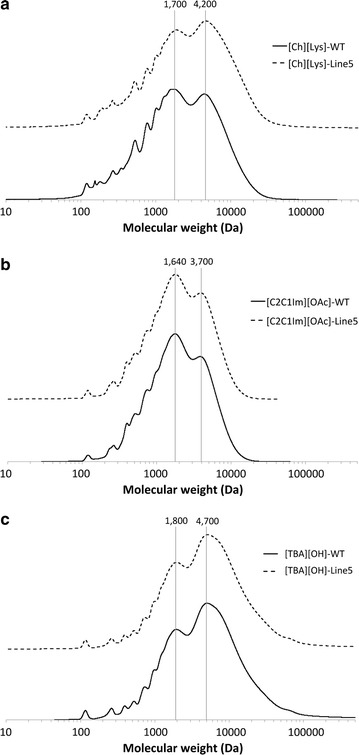
Gel-permeation chromatograms of lignin residue after enzymatic hydrolysis of (**a**) [Ch][Lys]-, (**b**) [C_2_C_1_Im][OAc]-, and (**c**) [TBA][OH]-pretreated poplar samples

Molecular weight distribution of solid residues isolated after enzymatic hydrolysis of poplar samples pretreated with the three ILs was measured to study the effect of zip lignin on the structural changes in the residual lignin fractions (Fig. 6). As shown, no significant differences were apparent regardless of the IL pretreatments. However, the average molecular weight of the lignin residue from the transgenic poplar was slightly higher than that from WT. For example, two distinctive peaks were found in the GPC profiles, and in the case of the [Ch][Lys]-pretreated transgenic poplar, the peak around 4200 Da was higher than that from the lower molar mass peak at ~1700 Da, whereas the WT showed the opposite trend. This can be explained by the fact that more weak linkages in the transgenic poplar were cleaved during pretreatment (see Table 2) and the fragments (because of the produced acid groups) were more soluble in the solvent (i.e., the lignins required less depolymerize to dissolve from the wall), whereas the more stable bonds remained. Table 2 shows the lignin content after IL pretreatments. As shown in Table 2, significant amount of lignin was removed after IL pretreatment, especially when pretreated with [Ch][Lys]. Lignin removals from Line 5 were considerably higher than that from WT regardless of IL used. These profiles likely illustrate the higher average molecular weight of lignin residue after enzymatic saccharification. In part, this is because the ferulates can actually participate in a greater variety of intractable linkages, while at the same time, introducing the readily cleavable ester bonds into the lignin backbone [4].

**Table 2:** Lignin content after IL pretreatments

	Lignin content after pretreatment (%)	Amount removed after pretreatment (%)
Raw [4]		
WT	20.2	–
Line 5	21.6	–
[Ch][Lys]		
WT	10.6	47.6
Line 5	7.9	63.4
[C_2_C_1_Im][OAc]		
WT	14.9	26.4
Line 5	12.0	44.4
[TBA][OH]		
WT	18.0	10.7
Line 5	16.5	23.7

When a comparison is made between three IL pretreatments, the average molecular weight of the solid residue was in the following order: [TBA][OH] ≫ [Ch][Lys] > [C_2_C_1_Im][OAc] in both WT and transgenic poplars. As previously discussed, both [C_2_C_1_Im][OAc] and [Ch][Lys] are highly capable of lignin removal during the pretreatment process [15, 20, 21, 33]. In addition, a higher pretreatment temperature is employed for these two ILs (compared to [TBA][OH]), and these conditions could be responsible for enhanced degradation of the lignin structure.

## Discussion

There have been many efforts to alter cell wall lignin content and composition in attempts to improve lignocellulosic biomass processing efficiency. Herein, we report on the IL pretreatment of engineered poplar lines in which monolignol ferulate conjugates were incorporated into lignins using a xylem-specific *CesA8* promoter driving an exotic *FMT* gene from Chinese angelica (*Angelica sinensis*) [4]. As previously reported, the contents of lignin in both WT and transgenic lines were not significantly different [4]. However, the amount of ML-FA conjugate in the lignins of transgenic poplars was higher (up to 13 times) than in WT, indicating that transgenic poplar lines have more chemically labile backbone linkages. Compositional analysis of IL-pretreated poplars resulted in lowered residual lignin contents from the transgenic poplar lines (see Additional file 1). Regardless of the ILs employed, the transgenic poplar lines consistently showed substantial improvements in saccharification yield after pretreatment. This indicates that increased sugar yield under limited-release conditions is correlated with lignin removal after IL pretreatment. [Ch][Lys] showed the highest improvement. [Ch][Lys] is known for its ability to dissolve lignin that can be removed from the materials to be saccharified by precipitation upon addition of an anti-solvent following the pretreatment step. ILs containing [Lys]^−^ anions, with their strong hydrogen bond basicity, have been found to provide an excellent delignification [15].

Although the glucose yield after enzymatic saccharification of [C_2_C_1_Im][OAc]-pretreated poplar was lower in the transgenic lines compared to that from [Ch][Lys] pretreated samples, its pretreatment efficacy has been well established [13, 20, 34, 35]. In general, [C_2_C_1_Im][OAc] pretreatment results in the disruption of inter- and intra-molecular hydrogen bonding between cellulose fibrils and lignin, resulting in solubilization of the biomass cell wall and substantially improving the pretreatment efficacy [13]. When pretreated with [TBA][OH], the yield of fermentable sugar was relatively low (~30%) compared to the other two ILs. Lower sugar yield could be attributed to the following: (1) some ILs have specific interactions with biomass, and those interactions are dependent on the cation, anion, temperature, and time used in the pretreatment step and (2) the extent and degree of biomass recalcitrance varies as a function of the biomass itself [2].

Overall, the strategic introduction of ester bonds into the lignin backbone in poplar produced biomass that can be processed more readily by IL treatments. This is a quite pleasing result as IL-base pretreatments are relatively biomass-agnostic; significant differences are expected for pretreatments that fare poorly for woody biomass, such as the ammonia-based processes, but apparently even processes such as IL pretreatment can benefit from the structural changes introduced into these transgenics. Among the transgenic lines, those with relatively high ML-FA conjugate levels in their lignins showed the highest digestibility. Equally important, the wood derived from the transgenic poplar requires lower chemical dosage and energy inputs for the production of fermentable sugars, which could improve the economic viability of ethanol production as well as biomass-derived value-added chemicals.

## Conclusions

The development of engineered biomass with reduced recalcitrance is a key technology that could improve the efficiency and viability of the next-generation biofuels. In this work, transgenic poplar that contains zip lignins, monolignol ferulate conjugate-derived units, was pretreated with ILs. The strategic introduction of ester bonds into the lignin backbone resulted in increased pretreatment efficiency and released more carbohydrates with lower energy input. The transgenic poplar lines showed higher levels of monomeric sugar release compared to the WT when treated with three different ILs and followed by enzymatic saccharification. These results clearly indicate that altering the natural lignification processes and taking advantage of its inherent plasticity has the potential to improve biomass conversion to liquid fuels. Combined with the development of lignocellulosic biomass streams that are more amenable to chemical depolymerization, IL pretreatment of biomass for enhanced biofuels production could facilitate a means to develop an economically viable and sustainable biofuel industry.

## Additional file

**Additional file 1: Figure S1.** Mass balances during three different IL pretreatments followed by enzymatic hydrolysis.

## Abbreviations

WT: wild type
IL: ionic liquid
ML-FA: monolignol ferulate
COMT: caffeic acid 3-*O*-methyltransferase
CCR: cinnamoyl-CoA reductase
FMT: feruloyl-CoA monolignol transferase
DI water: deionized water
HPLC: high-performance liquid chromatography
RI: refractive index
GPC: gel permeation chromatography
2D: two-dimensional
HSQC: heteronuclear single-quantum coherence
ANOVA: analysis of variance
