# Supplementary material for: Impact of lignin polymer backbone esters on ionic liquid pretreatment of poplar
Source: Biotechnol Biofuels. 2017 Apr 20;10:101. doi: 10.1186/s13068-017-0784-2 (PMC5399332; doi:10.1186/s13068-017-0784-2)
Supplement: Supplementary file 1 — Additional file 1: Figure S1. Mass balances during three different IL pretreatments followed by enzymatic hydrolysis. [file 13068_2017_784_MOESM1_ESM.docx]

**Additional file 1.**


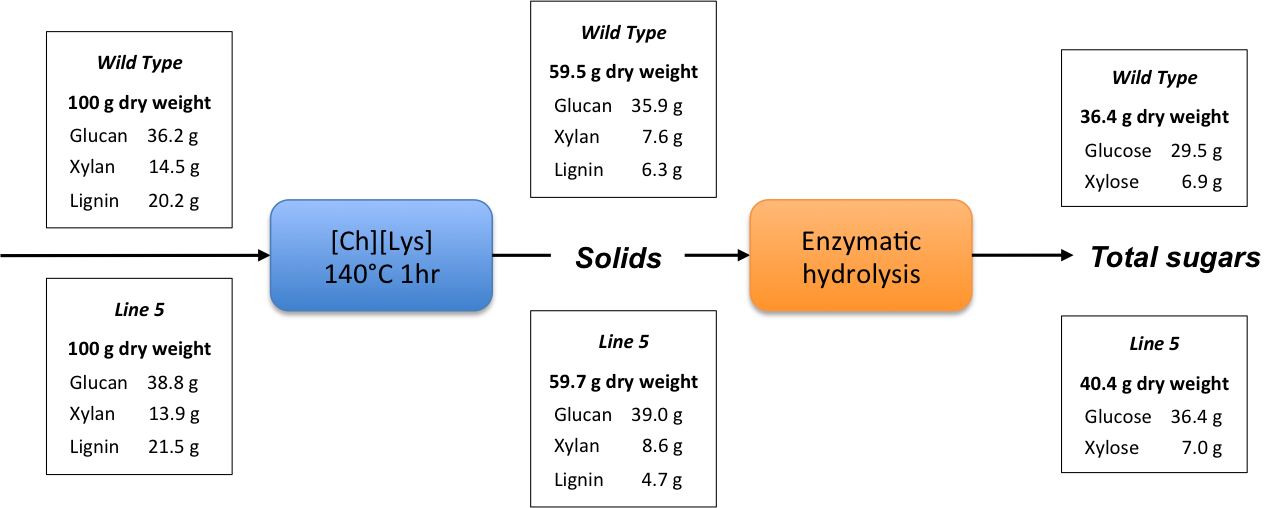


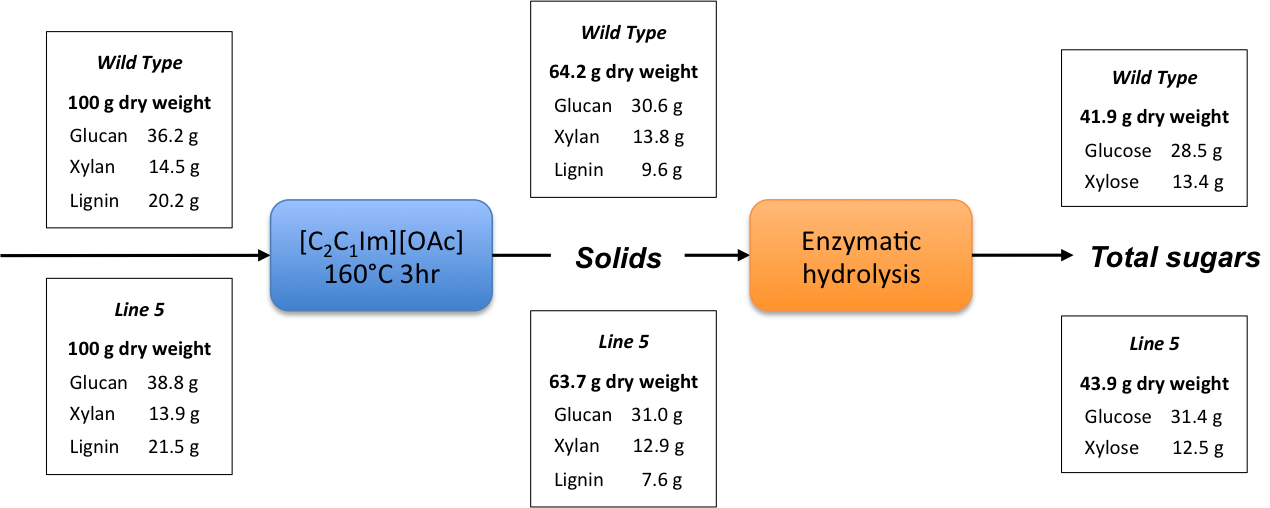


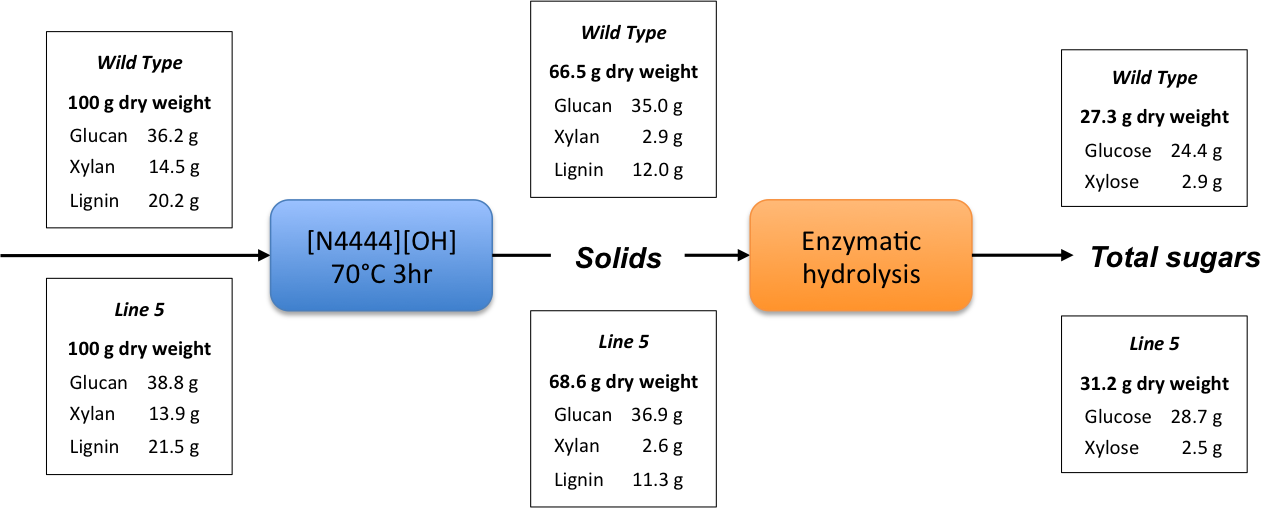


**Figure S1**. Mass balances during three different IL pretreatments followed by enzymatic hydrolysis.
